# A possible role for CCR5 in the progression of atherosclerosis in HIV-infected patients: a cross-sectional study

**DOI:** 10.1186/1742-6405-10-11

**Published:** 2013-05-09

**Authors:** Laura Fernández-Sender, Carlos Alonso-Villaverde, Anna Rull, Esther Rodríguez-Gallego, Marta Riera-Borrull, Anna Hernández-Aguilera, Jordi Camps, Raúl Beltrán-Debón, Gerard Aragonès, Javier A Menendez, Jorge Joven

**Affiliations:** 1 Unitat de Recerca Biomèdica, Hospital Universitari Sant Joan, Institut d’Investigació Sanitaria Pere Virgili (IISPV), Universitat Rovira i Virgili, Reus, Spain, carrer Sant Llorenç 21, Reus 43201, Spain; 2 Pressent address: Servei de Medicina Interna, Hospital Sant Pau i Santa Tecla, carrer Rambla Vella 14, Tarragona 43003, Spain; 3 Translational Research Laboratory, Catalan Institute of Oncology (ICO) and Girona Biomedical Research Institute (IdIBGi), Avda de França s/n 17007, Girona, Spain

**Keywords:** Cardiovascular risk factors, Chemokine receptors, Genetic variants, Inflammation, Maraviroc

## Abstract

**Background:**

Chemokines can block viral entry by interfering with HIV co-receptors and are recognised mediators of atherosclerosis development. A number of experimental drugs that inhibit HIV entry arrest the development of atherosclerosis in animal models. We hypothesised that the expression of chemokine receptors in circulating leukocytes is associated with the rate of atherosclerosis progression in HIV-infected patients.

**Methods:**

The increase in intima-media thickness during a 2-year follow-up was used to classify HIV-infected patients (n = 178) as progressors (n = 142) or non-progressors (n = 36) with respect to atherosclerosis. Logistic regression was used to assess variables associated with atherosclerosis progression. Mutations in the CCR5Δ32, CCR2 64I, and CX3CR1 (T280M and V249I) co-receptors as well as the levels of CCR5, CXCR4, CX3CR1, and CCR2 mRNA expression in circulating leukocytes were analysed as independent variables.

**Results:**

Among the baseline variables, only genetic variants explained the dichotomous outcome. The expression of CCR2 and CXCR4 did not discriminate between progressors and non-progressors. Conversely, CCR5 and CX3CR1 expression was higher in not only progressors but also patients with detectable viral load. The logistic regression, however, demonstrated a significant role for CCR5 expression as a predictor of atherosclerosis progression (B = 2.1, OR = 8.1, p = 0.04) and a negligible effect for CXC3R1 and CCR2 expression.

**Conclusions:**

Available CCR5 antagonists should be investigated for their potential to delay the course of atherosclerosis in HIV-infected patients.

## Background

Current therapies achieve long-term remission and/or prevent morbid conditions associated with human immunodeficiency virus-1 (HIV-1) infection. Despite the ineffective elimination of the virus, correct management is accompanied by a growing increase in life expectancy that involves age-related complications. For example, atherosclerosis and other non-communicable diseases develop earlier and more rapidly in HIV patients compared with non-infected individuals of a similar age with similar risk factors [1-3].

Persistent immune activation may be the reason for this phenomenon, likely through host gene variations in chemokine responses to non-infectious particles or viral proteins [4-6]. Chemokines and chemokine receptors play a significant role in atherosclerosis, may influence susceptibility to HIV infection, and may affect the course of both diseases [6,7]. The CC chemokines, which contain two adjacent cysteines near the amino terminus, are particularly important, and the role of CCL2, a ligand for CCR2, in the development of atherosclerosis has been substantiated in animal models and humans [5,8]. Furthermore, orally bioavailable agents that inhibit the chemotaxis of primary CCR2-expressing human monocytes towards CCL2 are currently in various clinical trials [9,10]. More recently, the chemokine receptor CCR5 and its ligands (CCL3, CCL4 and CCL5) have been implicated in the pathogenesis of atherosclerosis [11], and a relatively common mutation with a corresponding lack of function (CCR5 Δ32) confers relative resistance to HIV infection [12] and a reduction in susceptibility to coronary artery disease [13]. These and other findings have facilitated the development and further approval of CCR5 antagonists (maraviroc) as antiretroviral agents in patients infected with strains that do not use CXCR4 as the co-receptor [14]. It remains to be established whether maraviroc influences the development of atherosclerosis; however, we have recently identified the beneficial effects of both lipid metabolism and inflammation [15]. Fractalkine (CX3CL1) and its receptor, CX3CR1, have also been implicated as modulators of both HIV infection and atherosclerosis, and functional polymorphisms in CXC3R1 (V249I and T280M) are clinically relevant during the course of these conditions [16,17].

The available data are difficult to interpret because of the high redundancy of the chemokine system, conflicting results for the cardiovascular risk predictions, and effects from possible interactions among receptors, which could be confounding factors [16,17]. In this study, we investigated for the first time whether the expression of chemokine receptors in circulating leukocytes was relevant for the assessment of atherosclerosis progression in HIV-infected patients. We determined that CCR5 expression in these cells might be a strong predictor of atherosclerosis progression, indicating a further therapeutic target for CCR5 antagonists.

## Methods

### Study design, population and standardised ultrasound protocol

This report was prepared in accordance with the STROBE guidelines described at http://www.strobe-statement.org/. The procedures were performed in accordance with the Code of Ethics of the World Medical Association (Declaration of Helsinki) for experiments involving humans, and the study was approved by the ethics committee at the Hospital Universitari de Sant Joan de Reus. Patients who accepted the invitation to participate in the study provided full informed consent. HIV-infected patients who attended our outpatient clinic were routinely asked to be incorporated into a cohort (n = 523) in accordance with previously described procedures [1,6,18]. None of the included patients were treated with CCR5 antagonists. In a retrospective, longitudinal study, we sampled a cross-section of the patients to assess the intima-media thickness (IMT) at baseline (when incorporated into the cohort) and 2 years later.

We used the LOGIQ 700 ME system (General Electric) to obtain normalised values IMT [1,6]. We identified and digitally recorded the far wall of the common carotid artery (1 cm proximal to the bifurcation), the carotid bulb (in the bifurcation), and the internal carotid artery (1 cm distal from the bifurcation). The IMT measurements of each arterial segment were averaged and used in the statistical analyses as the combined IMT. We identified patients with a 2-year difference in the IMT value that was equal to or lower than the technical differences among examiners as non-progressors. This value was obtained via an intraclass correlation coefficient determined with 30 measurements (r = 0.89, p < 0.001) and established as 0.01 mm (standard deviation 0.02 mm). The progressors exhibited ΔIMT >0.01 mm and/or the presence of a previously unrecognised plaque in the second observation. The procedures were performed essentially as described previously [6,19,20]. To assess the reproducibility of the measurements, the images of 30 randomly selected patients were re-measured using the same protocol. The intraclass correlation coefficient between the 2 sets of measurements was 0.91, and the absolute difference in IMT was 0.007 mm (0.018). To assess the reproducibility of the rescanning and re-measurement, we also measured the inter-month correlation (r = 0.95; n = 15).

The other relevant data were collated from clinical records or obtained using standardised guidelines and routine laboratory methods [21,22]. The covariates used were obtained in the same time frame for each participant. To avoid sampling bias, we selected and presented data only from patients who did not exhibit changes in variables or episodes that were likely to contribute to the outcome during follow-up (n = 217). The patients were observed by their attending doctors every 3 months, and no major events were recorded. Changes in the clinical and laboratory markers of cardiovascular risk and/or HIV infection were considered not relevant (less than 5% of differences with respect to the normal inter-day coefficient of variation for each measurement). The resulting study population was used to determine whether chemokine receptor expression in blood cells was associated with atherosclerosis progression, and additional fasting venous blood samples were drawn immediately before the second IMT measurement under conditions that guaranteed RNA preservation (using TEMPUS blood RNA tubes from Applied Biosystems, Foster City, CA). Inappropriately documented IMT measurements (n = 16), inappropriately handled samples (n = 9), and changes in the antiretroviral treatment (n = 14) were detected and considered exclusion criteria. Therefore, the data for 178 patients are presented. This number was considered sufficiently higher than the minimum sample size obtained (n = 121) according to available data [6,19,20] and assuming a difference between groups exceeding 10%, an overall standard deviation of 20%, and 80% power with a two-sided 5% significance level.

### DNA genotyping and mRNA expression

The methods used to isolate DNA and assess the presence of CCR-2 V64I, CCR-5 Δ32, and CX3CR-1 (V249I and T280M) variants have been described in detail [6,23] (see Additional file 1: Table S1 for additional information). RNA in circulating leukocytes was isolated using an ABI PRISM 6100 (Applied Biosystems), and the expression of selected receptors (CCR5, CCR2, CXCR4 and CX3CR1) was measured using the reagents and methods indicated in Table 1. The results were analysed using RQ Manager Software for automated data analysis. The expression values for the target genes were normalised to the values of a designated endogenous control (GAPDH), which was not affected by the HIV infection in our hands, and expressed in arbitrary units [24,25]. We hypothesised that there may be differences between the distribution of the mRNAs and the localisation of the actual proteins and that it was unlikely that surface cell receptors could be delivered to other tissues or undergo posttranslational rearrangements [26].

**Table 1:** Summary of taqman® gene expression assays used in this study*

**Gene symbol**	Assay ID*	**Context sequence**	**Amplicon length (bp)**
CX3CR1	Hs00365842_m1	CAGTCCACGCCAGGCCTTCACCATG	84
*CCR2*	Hs00174150_m1	GGGGAGAAGTTCAGAAGCCTTTTTC	74
*CXCR4*	Hs00607978_s1	CCTGTCCTGCTATTGCATTATCATC	153
*CCR5*	Hs00152917_m1	GAAACTCTCCCCGGGTGGAACAAGA	98
*GAPDH*	Hs99999905_m1	TTGGGCGCCTGGTCACCAGGGCTGC	124

*Thermal cycling and fluorescence detection was performed on the Applied Biosystems ABI Prism 7900HT Detection System with ABI Prism 7900HT SDS Software 2.2. Forty cycles were run with the following parameters: 2 mins at 50°C and 10 mins at 94.5°C and for each cycle 30 secs at 97°C for denaturation and 1 min at 59.7°C for transcription. FAM was the reporter dye.

### Statistical analyses

The data were presented as the means plus the standard error of the mean (in parentheses). The normality of the distributions was assessed using the Kolmogorov-Smirnov method. The groups were compared using Student’s unpaired *t*-test or Kruskal–Wallis one-way analysis. There were no adjustments for multiple testing for genetic analyses because the variants were not rare and the correspondence for their phenotypes was absent according to the described procedures [27]. The χ^2^ test was used to compare categorical variables. Multivariate logistic regression stepwise analyses were performed to detect factors associated with the condition of progressors. Odds ratios were included when necessary. Allelic frequency descriptions and Hardy-Weinberg equilibrium tests were performed using freely available software (http://bioinfo.iconcologia.net/SNPstats). To create a “quasi-randomised” experiment, we used the propensity score analyses described previously [28-30]. For other measurements, we used SPSS software version 18.0 (SPSS Inc., Chicago, IL).

## Results and discussion

In accordance with our expectations, most of the patients included in the study were considered progressors (n = 142); however, a substantial number of patients demonstrated no evidence of increased IMT values during the 2-year study (n = 36). The patients who demonstrated progression of subclinical atherosclerosis were treated with either an efavirenz-based regimen (38%) or protease inhibitors (39%), and a number of patients were left untreated. The majority of the patients maintained a low viral load. The treatment regimens for patients who did not demonstrate progression were similar. We did not observe a between-group difference in the lipid-lowering and/or hypoglycaemic treatments, and to avoid confusion, none of the patients were taking CCR5 antagonists.

The treatment and other baseline clinical and laboratory variables (Table 2) did not allow the prediction of whether subclinical atherosclerosis would progress. However, this does not mean that any of these variables could be determinants of progression because according to our study design, only negligible changes were allowed. We appreciate that this is not feasible for normal clinical management procedures; however, we believe it would be helpful to search for other possible predisposing factors. Intervention and randomisation were not used in this study. The presence of bias was considered unlikely but could be a limitation of the study as discussed below. Despite these considerations, we reasonably excluded the robust influence of factors such as age, gender, smoking habits, lipoprotein disturbances, the presence of lipodystrophy (fat loss or accumulation perceived by the patient or the clinician) or HCV co-infection, inflammation and insulin resistance (Table 2).

**Table 2:** Baseline participants’ clinical and laboratory characteristics blindly collated from medical records at the end of the study

	**Non-progressors (n = 36; 20%)**	**Progressors (n = 142; 80%)**	**p value**
Age, years.	42.1 (1.5)	42.3 (8.2)	NS
Male, n (%)	22 (61.1)	105 (73.9)	NS
BMI, kg/m^2^	22.9 (0.5)	23 (3.2)	NS
Current smokers, n (%)	27 (75.8)	92 (64.6)	NS
Systolic blood pressure, mm Hg	116 (2)	123 (2)	NS
Diastolic blood pressure, mm Hg	77 (1)	78 (1)	NS
Baseline IMT, mm	0.81 (0.03)	0.75 (0.02)	NS
Final IMT, mm	0.81 (0.02)	0.94 (0.02)	<0.0001
Δ IMT, mm	0 (0.01)	0.19 (0.02)	<0.0001
Total cholesterol, mmol/L	4.62 (0.21)	4.63 (0.14)	NS
HDL cholesterol, mmol/L	1.21 (0.05)	1.12 (0.04)	NS
LDL cholesterol, mmol/L	2.44 (0.14)	2.54 (0.12)	NS
Triglycerides, mmol/L	2.42 (0.33)	2.31 (0.29)	NS
Apolipoprotein A I, mmol/L	1.42 (0.04)	1.45 (0.04)	NS
Oxidized-LDL, ng/mL	86.4 (5.3)	98.6 (12.7)	NS
Lipodystrophy, n (%)	23 (63.9)	101 (71.1)	NS
HVC co-infection, n (%)	24 (66.7)	77 (54.2)	NS
hs-CRP, mg/L.	4.4 (0.7)	3.8 (0.7)	NS
Insulin, pmol/L	81.2 (10)	71 (8)	NS
Glucose, mmol/L.	5.4 (0.2)	5.2 (0.4)	NS
HIV-1 RNA < 50 copies/mL, n (%)	28 (77.2)	110 (77.5)	NS
CD4 cell count, cell/mm^3^	575 (45)	485 (37)	NS
CD8 cell count, cell/mm^3^	1020 (65)	994 (55)	NS
Δ CD4 cell count, cell/mm^3^	37 (36)	36 (31)	NS

BMI, body mass index; HDL, high density lipoprotein; IMT, intima-media thickness; LDL, low density lipoprotein. Propensity scores calculated via regression analyses were close to 0 indicating that the probability of these variables affecting the progression in IMT is low.

Arguably, a 2-year follow-up might not be sufficient to evaluate the full effect of the variables. A longer follow-up to ascertain the role of host-related, genetically determined variants, however, is not feasible because the progression of carotid atherosclerosis in these patients is faster than in non-infected patients, and the evaluation of non-classical factors requires shorter periods [19,20]. We have previously demonstrated non-concordance between subclinical atherosclerosis and the calculated Framingham risk score in HIV-infected patients [31]. However, the issue of non-concordance is under debate and in a recent study, increased Framingham risk scores were associated with abnormal early and late surrogate markers of atherosclerosis. However, low scores were also associated with significant subclinical atherosclerosis in these patients [32].

**Figure 1:**
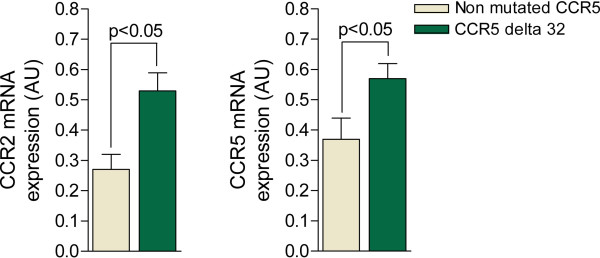
**The presence of the CCR-5** Δ**32 polymorphism in HIV-infected patients was associated with a significant increase in the expression of CCR2 and CCR5 in circulating leukocytes with respect to patients without the mutation.** Similarly, the CCR2 V62I was also associated with a similarly higher CCR5 expression (data not shown).

Despite the identification of several trends (Table 3), the distribution of selected chemokine-related genetic variants did not demonstrate significant differences between the progressors and non-progressors. Next, we measured the mRNA expression of chemokine receptors involved in HIV entry into leukocytes. The chemokine receptors were selected according to available data implicating these receptors in the course of atherosclerosis. Interestingly, we observed that the variant CCR5 Δ32 was responsible for the increased expression of these receptors, but only CCR2 and CCR5 reached statistical significance (Figure 1). Patients who expressed the CCR2 V62I gene variant also demonstrated a two-fold higher expression (p = 0.004) of CCR5 compared with non-carriers (data not shown)*.* The expression of chemokine receptors, especially CCR5, did not correlate with increases in IMT. However, CX3CR1 expression demonstrated a marginal effect (ρ = 0.21, P = 0.053). The lack of significant correlations does not discard a quantitative relationship because the influence of high variability and the absence of normal distribution may mask the association between continuous variables.

**Table 3:** Allelic frequency for selected polymorphisms as segregated according to the progression in IMT measurements during a two-year period

**Allele**	**Non-progressors**	**Progressors**	**P-value**
**CXC3CR-1 249 V**	0.61	0.50	NS
**CXC3CR-1 280 M**	0.10	0.12	NS
**CCR2 62 I**	0.07	0.08	NS
**CCR5 Δ32**	0.03	0.08	0.08

**Figure 2:**
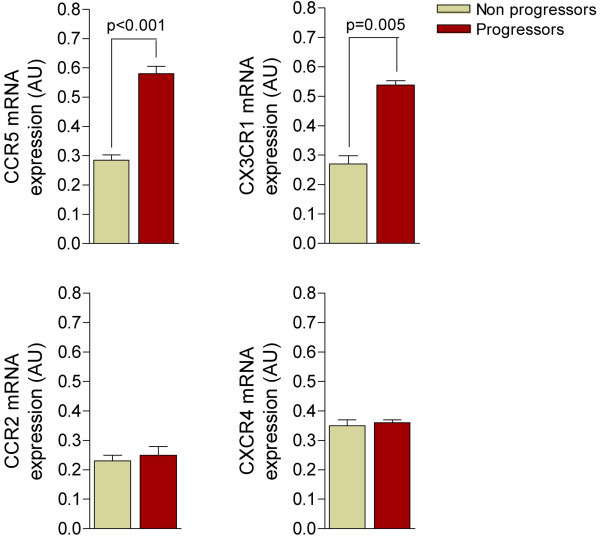
The expression of CCR2 and CXCR4 in circulating leukocytes from progressors and non-progressors was similar but there were a significant increase in CCR5 and CX3CR1 in patients who showed progression in intima-media thickness.

**Figure 3:**
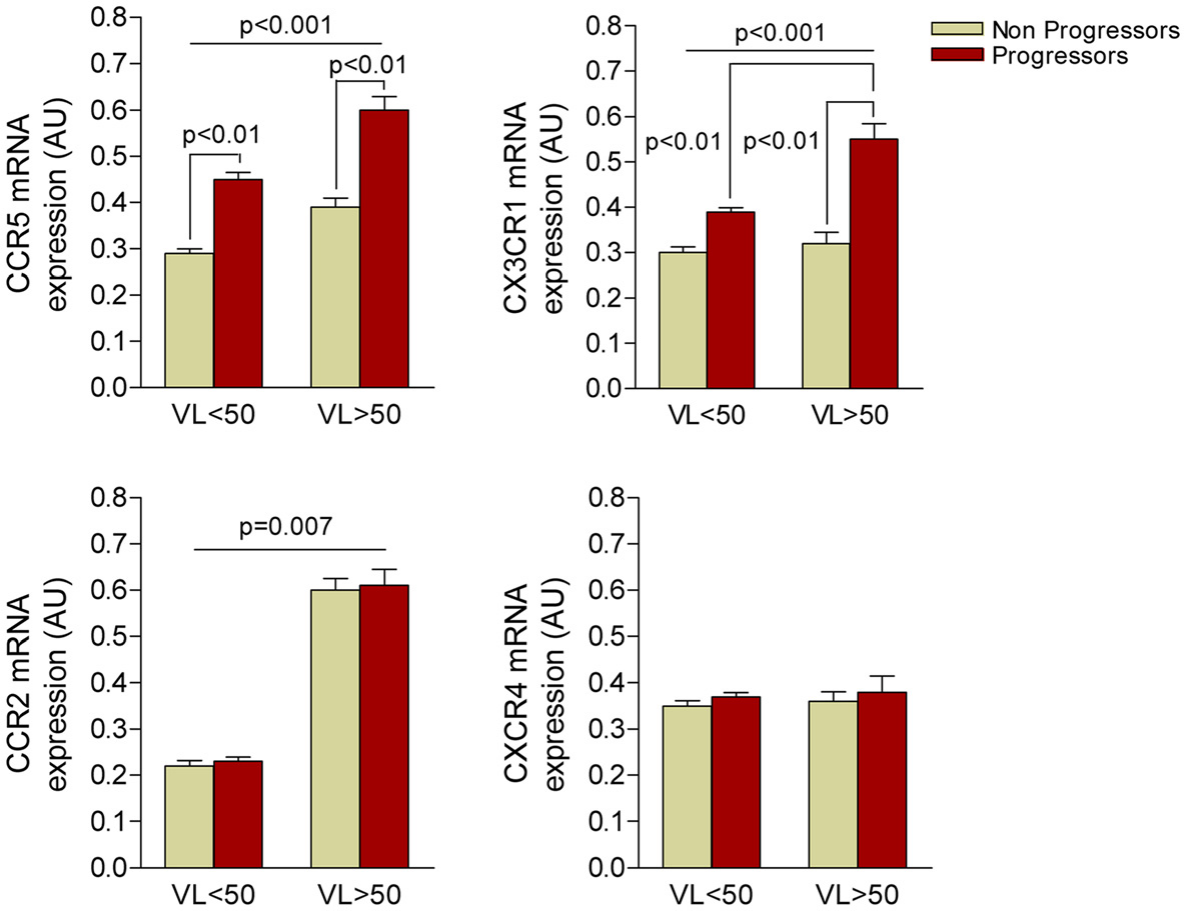
**Detectable viral load (VL) was also a determinant factor in the rate of expression of chemokine receptors with the exception of CXCR4.** The expression of CCR2 was higher in patients with detectable viral load but this difference was not related to the progression of atherosclerosis. Conversely, the increase in CCR5 and CX3CR1 expression was not only higher in progressors but also in those with detectable viral load.

We observed that the expression of CCR2 and CXCR4 did not discriminate between progressors and non-progressors, and the expression of CCR5 and CX3CR1 was significantly higher in the progressors (Figure 2). These results are plausible because the data obtained in humans and animal models indicate that CCR5 is crucial for monocyte recruitment, and CX3CR1 appears to sustain chronic monocyte adhesion and survival within the plaque in diseased arteries during atherosclerosis development [33,34]. Because the increase in CCR5 and CX3CR1 expression was higher in not only progressors but also the patients with detectable viral load, our data might support a role for the virus in determining the rapid development of atherosclerosis [35,36]. No differences were found in CXCR4; however, the expression of CCR2 was 3-fold higher in patients with a detectable viral load (p = 0.007), an effect that was considered independent of the condition of either progressors or non-progressors (Figure 3). Therefore, further studies will be needed to ascertain a relationship between relatively poor management of the infection and the development of vascular lesions. This interpretation is substantiated by the observation that a number of chemokines block or delay viral entry by interfering with HIV co-receptors [37]. There are multiple plausible mechanisms underlying the interference between the retroviral life cycle and chemokines. First, endothelial cells line the entire cardiovascular system and are frequently in contact with memory CD4+ T cells, which provide signals to HIV-1-infected CD4+ T cells to enhance HIV-1 production, an effect that persists despite antiretroviral therapy [36]. Second, infiltrating HIV-infected monocytes/macrophages promote proinflammatory effects *per se*. This process is important because HIV itself can infect vascular smooth muscle cells via a mechanism dependent on chemokine receptors, and this may partially explain the exacerbated atherosclerosis reported in infected individuals [37].

**Figure 4:**
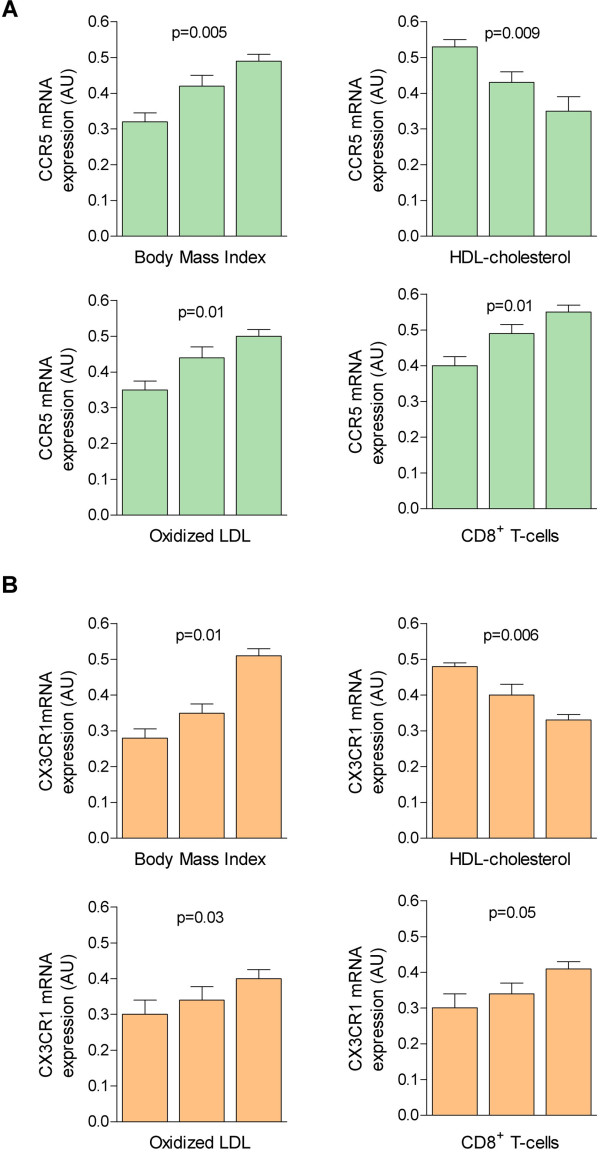
**Chemokine receptors and selected variables.** The expression of CCR5 (**A**) and CX3CR1 (**B**) are affected by modifiable factors and increased in patients with the highest values of body mass index, the concentration of oxidized LDL-ox, and the number of circulating CD8^**+**^ T cells and the lowest value for HDL-cholesterol. The cutoff values for the first and second tertiles were respectively: BMI, 20.1 and 23.3; HDL-cholesterol, 0.84 and 1.11 mmol/L; ox-LDL, 51.1 and 80.2 ng/mL, and CD8, 592 and 920 cells/mm^3^.

The course of HIV infection, however, may also be associated with other factors, such as body mass index (BMI), high-density lipoproteins (HDLs) or reverse cholesterol transport from macrophages, the amount of circulating oxidised low-density lipoproteins (LDL-ox) and the number of CD8^+^ T cells in the blood [38-40]. We investigated this possibility and determined that when the values of these variables were divided into tertiles, a qualitatively similar effect on the expression of CCR5 and CX3CR1 was observed (Figure 4). The expression of both receptors was higher in patients with the highest BMI, LDL-ox and CD8^+^ T cells. Additionally, patients with the lowest value for HDL cholesterol demonstrated a higher increase in CCR5 and CX3CR1 expression. These findings support the hypothesis that several modifiable factors cannot be excluded in the assessment of a possible association between the course of accelerated atherosclerosis and the course of HIV infection [31-33,41-43]. Univariate analyses suggested that these assumptions were correct, and multivariate logistic regression analyses determined that the dichotomous model was explained by only a small number of variables (Table 4). Logistic regression was used rather than discriminant function analysis to include predictors lacking normal distribution, and all predictors were used to determine group membership according to outcome (progressors and non-progressors).

**Table 4:** Significant predictor variables found in multivariate logistic regression stepwise analysis used for the binary outcome (progressors and non-progressors) with respect to subclinical atherosclerosis

	**B**	**Wald**	**p value**	**Odds ratio**	**CI 95% for OR**	
					**Lower**	**Upper**
**CCR5 expression**	2.1	3.9	0.04	8.1	1	65
**CCR5 Δ32**	−2.2	4.9	0.02	0.1	0.01	0.71
**CX3CR1 280 V**	−2.9	7	0.008	0.05	0.005	0.462
**CX3CR1 249 M**	−1.4	3.5	0.05	0.24	0.05	1.05

Generally, we found that CCR5 expression is the best predictor of progression in atherosclerosis and that a number of host gene variants may have a significant effect. Although CCR2 and CX3CR1 are predisposing factors in atherosclerosis and inflammation [41,42], we also determined that under these conditions, the effects of the expression of these receptors appeared negligible. This is not surprising because the expression of chemokines in different tissues can change in response to multiple stimuli [44].

The predominant role of CCR5 expression in circulating leukocytes in HIV-infected patients as a predictive factor for the rate of progression of atherosclerosis suggests that drugs affecting chemokines should be investigated for their role not only as protective agents in HIV-infection but also in atherosclerosis-related complications. This suggestion is in accordance with the observation that a number of experimental drugs that inhibit HIV entry can arrest the progression of atheroma plaques in animal models [45-47] and that CCR5 antagonists can affect cardiovascular risk factors [48,49]. Therefore, we propose that available CCR5 antagonists that have been clinically validated should be considered as not only antiviral drugs but also potentially useful agents for the management of inflammation-related diseases.

### Possible limitations of the study

Biases are particularly difficult to avoid in studies with these characteristics. However, in this study, recall bias and detection bias were essentially excluded from the design. We also excluded the possibility that the increase in the IMT measurement was sensitive to small, unmeasured confounders (hidden biases) because the patients were examined at least 8 times during the study according to a strict clinical protocol for the detection of the outcome and related variables. Additionally, covariate adjustment using propensity score analyses demonstrated good performance, with scores reflecting a low probability that the participant’s baseline characteristics played a role in the increase in IMT. Finally, we appreciate that the length of the follow-up may be important and should be considered carefully when replicating our findings.

## Competing interests

The authors declare that they have no competing interests and have no conflict of interest to declare.

## Authors’ contributions

All authors have contributed to the acquisition, analysis and interpretation of data and have been involved in drafting and revising the manuscript. The design was initially conceived by C A-V, L F-S and JJ. All authors have given final approval of the version to be published and may take public responsibility of the content.

## Supplementary Material

Additional file 1: Table S1 Summary of gene polymorphisms genotyped in this study. Click here for file
