# Supplementary material for: A possible role for CCR5 in the progression of atherosclerosis in HIV-infected patients: a cross-sectional study
Source: AIDS Res Ther. 2013 May 9;10:11. doi: 10.1186/1742-6405-10-11 (PMC3663662; doi:10.1186/1742-6405-10-11)
Supplement: Additional file 1: Table S1 — Summary of gene polymorphisms genotyped in this study. [file 1742-6405-10-11-S1.doc]

| Supplementary Table 1 Summary of gene polymorphisms genotyped in this study | | | | |
| --- | --- | --- | --- | --- |
| **SNP ID** | **Gene Name** | **SNP description** | **AB Assays-on-Demand^TM^ SNP Genotyping Assay** | Taqman probes*[VIC/FAM] |
| rs333 | CCR5 | Δ32 | Not available | Genomic DNA was amplified with primers flanking the 32 bp deletion, and PCRs were electrophoresed on 3% agarose gels to visualize the wild-type and Δ32 alleles. |
| rs1799864 | *CCR2* | V64I | Not available | DNA was amplified with primers 5′ATG CTG TCC ACA TCT CGT TCT (forward) and 5′TTT TTG CAG TTT ATT AAG ATG AGT (reverse). The 215 bp PCR-product was digested with the restriction enzyme RsaI, and after electrophoresis on 3% agarose gels the two alleles were visualized as fragments of 215 bp (64 I) and 191 + 24 bp (64 V). |
| rs3732379 | *CX3CR1* | T280M | C_7900503_1 | TTAAGCGTCTCCAGGAAAATCATAA[C/T]GTTGTAGGGTGTCCAGAAGAGGAAA |
| rs3732378 | *CX3CR1* | V249I | C_5687_1 | CAGGCAACAATGGCTAAATGCAACC[A/G]TCTCAGTCACACTGAGGGCCAGCCT |
| * Allelic discrimination was performed using 20 ng of DNA in a 5 μL reaction mixture containing 2.5 μL TaqMan Universal polymerase chain reaction mix, 0.25 μl TaqMan SNP Genotyping Assay, and 2.25 μl of DEPC-treated water. Reaction conditions consisted of preincubation at 50°C for two minutes and 95°C for ten minutes, followed by 40 cycles of 95°C for 15 seconds and 60°C for 1 minute. Amplifications were performed in a 7900HT Sequencing Detection System for continuous fluorescence monitoring. | | | | |
